# Prevalence of *Helicobacter pylori* among Patients with Gastrointestinal Tract (GIT) Symptoms: A Retrospective Study at Selected Africa Air Rescue (AAR) Clinics in Kampala, Uganda, from 2015 to 2019

**DOI:** 10.1155/2021/9935142

**Published:** 2021-11-08

**Authors:** Edity Namyalo, Luke Nyakarahuka, Matthias Afayoa, Joel Baziira, Andrew Tamale, G. Collins Atuhaire, Joseph M. Kungu

**Affiliations:** ^1^College of Veterinary Medicine Animal Resources and Biosecurity, Makerere University, Kampala, Uganda; ^2^Department of Microbiology, Mbarara University of Science and Technology, Kampala, Uganda

## Abstract

**Background:**

*Helicobacter pylori* (HP) infection is extremely common worldwide, with almost half of the world's population infected. In Uganda, no study has been done on the trends of the prevalence of *H*. *pylori* infection in the affluent population. Therefore, this retrospective cross-sectional study aimed at determining the trend of *H*. *pylori* prevalence among affluent patients presenting with gastrointestinal (GIT) symptoms whose stool samples were tested at selected AAR clinics in Kampala area. Patients were tested for *Helicobacter pylori* infection using the stool antigen test between January 2015 and December 2019.

**Results:**

The overall 5-year *H. pylori* prevalence was 35.7% (1298/3634). The prevalence was higher in males (36.0% (736/2044)) than in females (35.4% (562/1590)), although not statistically significant (OR = 0.97, *p* = 0.680, 95% CI: 0.84–1.11). The prevalence of *H*. *pylori* infection was significantly higher (39.4%) among patients who belonged to the age group of 19–35 years (OR = 1.49, *p* < 0.001, 95% CI: 1.22–1.82). The prevalence for *H*. *pylori* among the age group of 19–35, the most productive age, could be attributed to work-related factors such as stress. The highest prevalence (43.4%) was recorded in 2018 and the lowest (21.4%) in 2015; however, the trend of *H*. *pylori* infection in the 5 years was fluctuating.

**Conclusion:**

*H*. *pylori* infestation is a preserve of not only the poor but also the elites. Stressful factors, especially in the age group of 19–35 years, should be appropriately managed.

## 1. Introduction

*Helicobacter pylori* (HP) infection is very common worldwide with evidence from a recent systematic review suggesting that almost half of the world's population is infected [1, 2]. This bacterium was first discovered in 1982 in stomach specimens of patients with gastritis and peptic ulceration [3].

*Helicobacter pylori* is a motile Gram-negative bacillus, corkscrew shaped, oxidase negative, catalase negative, urease positive, and microaerophilic bacillus [4]. Being microaerophilic has greatly contributed to its survival in the stomach because it needs only about 4% oxygen, 5% carbon dioxide, and 5% hydrogen for its growth and survival. It is very motile because it has multiple flagella that emerge from one of the rounded ends [4].

*Helicobacter pylori* infection is characterized by symptoms such as heartburn, belly pain or swelling, nausea, unexplained anaemia, not feeling hungry, feeling full after you eat just a small amount, vomiting, and weight loss for no reason.

In 1994, the International Agency for Research on Cancer (IARC) defined *H*. *pylori* as a group 1 carcinogen, and yet, there are no effective therapies for gastric cancer and other cancers caused as a result of poor management of this bacterium. This has contributed to increased morbidity and mortality rates, more so the growing pattern of antibiotic resistances is further complicating the treatment of *H*. *pylori* infection [5].

Globally, infection with *H*. *pylori* is associated with several upper gastrointestinal diseases including gastritis, dyspepsia, peptic ulcer, duodenal ulcers, and gastric cancers such as mucosa-associated lymphoid tissue (MALT) lymphoma and gastric adenocarcinoma. In addition, idiopathic thrombocytopenic purpura and iron deﬁciency anaemia have also been associated with *H*. *pylori* infection [6, 7].

It has been documented that infection rates are higher in resource-poor settings and developing countries, with prevalence rates above 70% reported in Africa, the highest worldwide [2], whereas developed countries range between 25 and 40% [2, 8]. The high prevalence in developing countries has been associated with low socioeconomic status, overcrowding, poor housing, poor sanitation (both personal and environmental hygiene), unclean water supplies, accumulation of animal faecal matter, and food contamination [9–11].

Many studies from Africa have shown high prevalence, for example, 81.7% in Nigeria [12], 53.0% in Egypt [13], 39.1% in Tanzania [14], 64.39% in Cameroon [15], and 73.3% (among children) and 54.8% (adults) in Kenya [16].

There are a few *H*. *pylori* prevalence studies that have been done in Uganda; for example, studies in Kampala have reported a prevalence of 87% among patients with stomach cancers and other cancers [17], 44.3% among children between 0 and 12 years [18], and 60.5% among pregnant women at Kawempe Health Centre [19].

Poor management of *H*. *pylori* infection can contribute to the occurrence of cancers of various organs such as stomach, duodenum, pancreas, and liver cancer especially in patients coinfected with hepatitis viruses; acceleration of the disease progression has been observed [20]. Gastric cancer is among the five most fatal types of cancer, and according to the statistics of the World Health Organization (WHO), about 783,000 patients die each year after developing the disease [21].

In Uganda, particularly in divisions of Kampala city, few studies have been done on the prevalence of this common infection especially in corporate clinics such as Africa Air Rescue (AAR). These clinics provide medical services to mostly middle-income and high-income classes of people. Regardless of economic status, *H*. *pylori* infection effects are adverse or even irreversible when poorly managed [22]. However, there is still scanty information on trends and burden of *H*. *pylori* in urban areas such as Kampala especially among the elite people that seek medical care at AAR clinics and hence the need of this study.

## 2. Methods

### 2.1. Study Area

**Figure 1:**
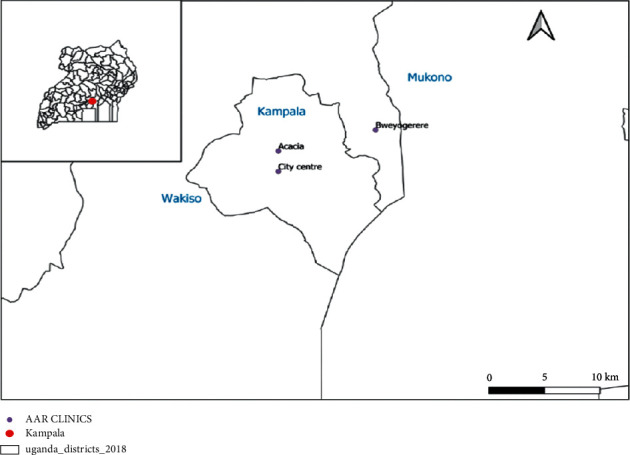
A map showing the location of study sites (inset is the map of Uganda). Note: this map was generated in QGIS as it cannot be changed to other editable formats.

The study was carried out at three randomly selected AAR clinics located in Kampala district, central region of Uganda. Kampala is the largest city with the highest population of 1,507,080 million [23] and has a combination of people with various socioeconomic status, level of education, living conditions, densely populated, and various water sources for routine home use. The selected clinics include City Centre Clinic which is along Parliamentary Avenue, Acacia Clinic located in Kololo, Makindu close, and Bweyogerere Clinic which is along Kampala-Jinja Highway as shown in Figure 1. These clinics mainly offer outpatient clinical services to both insured and private clients; however, the biggest proportion of their clients was insured.

### 2.2. Study Design

A retrospective cross-sectional study was carried out for a period of five years, from January 2015 to December 2019, at randomly selected AAR (Africa Air Rescue) clinics which had been in existence for five or more years in Kampala district.

### 2.3. Study Population

The study population included all clients that presented with GIT symptoms and carried out an *H*. *pylori* stool antigen test during the five years of the study regardless of the age.

### 2.4. Inclusion and Exclusion Criteria

Only GIT patients who were tested for *H*. *pylori* infection using the stool antigen test were included in the study regardless of the age and sex. GIT patients who had completed treatment below three months of diagnosis and tested for *H*. *pylori* were excluded.

### 2.5. Sampling Strategy

**Figure 2:**
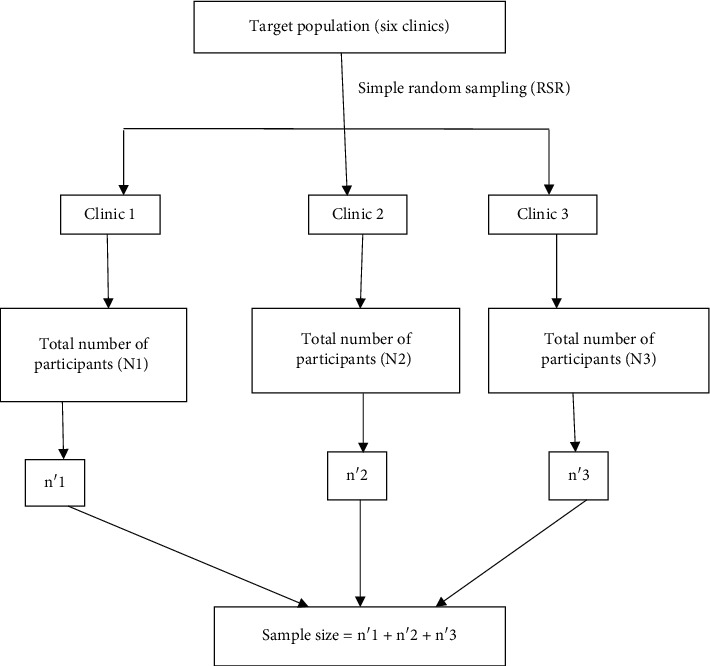
Multistage sampling frame.

AAR Healthcare has a total of six clinics (sampling units) (Acacia, Kabalagala, Makerere, City Centre, Bugolobi, and Bweyogerere) that had existed for more than five years in Kampala area; out of these, three clinics were selected using simple random sampling. The six clinics were assigned numbers from 1 to 6, these were put into the Microsoft Excel sheet, and then random numbers were selected. The stratified random sampling using the proportional allocation method was used on each stratum to obtain the sample size which was used to estimate the prevalence of *H*. *pylori*. Details of the sampling strategy are shown in Figure 2.

### 2.6. Data Collection and Analysis

All patients that met the inclusion criteria were included in the study regardless of their age. Examination and *H*. *pylori* SAT test results were obtained from the clinic information system (Paras system) from 2015 to 2019. A stool antigen test was chosen rather than a serology antibody test because it is more specific and it detects only active infection since it is an antigen test. Data collected were entered and cleaned in the Microsoft Excel sheet, and then statistical analysis was performed using Stata version 14.2. Data were summarized and tabulated in Stata to generate the general annual, monthly, and clinic-based prevalence. The generated data were presented as graphs to depict trends of *H*. *pylori* infection among patients that visited AAR clinics between 2015 and 2019. Bivariate logistic regression analysis was performed to test for associations between *H*. *pylori* infection and age, sex, and location.

### 2.7. Laboratory Diagnosis of *H*. *pylori*

There are various methods of testing *H*. *pylori* which can be invasive and noninvasive. These include urea breath test (UBT), stool antigen test (SAT), serological tests, endoscopy, histology, culture, and polymerase chain reaction (PCR). This study considered the SAT technique.

A stool sample was collected in a sterile container, and 50 mg of stool (equivalent to ¼ of a pea) was mixed with the buffer. Three full drops (120 *μ*L) of the specimen were put into the sample well of the cassette. The positive result could be read as early as 5 minutes; however, negative results were confirmed after 15 minutes. This SAT technique was preferred for this study because it is more specific than the blood screening test and has sensitivity and specificity of 73.9% and 86.7%, respectively [12].

### 2.8. Sample Size Estimation

The sample size was determined using an established formula [24]:

(1) $$\begin{array}{c} n=\frac{Z^{2}PQ}{D^{2}}. \end{array}$$

The calculated minimum sample size for such a study is 384 patients, but since this is a 5-year retrospective study, a bigger number of 3634 patients was used to increase precision.

## 3. Results

### 3.1. Demographic Distribution of the Patients

A total of 3,634 patients were included in this study, with 56.3% (2044/3634) male and 43.75% (1590/3634) female. The mean age of all participants was 32.4 years (SD = 0.227, 95% CI: 32.0–32.9), and median was 33 years. Acacia Clinic contributed the highest proportion of patients of 52.8% (1918/3634), followed by City Centre Clinic with 26.1% (950/3634) and Bweyogerere Clinic (BC) with 21.1% (766/3634). Different age categories were considered in this study, that is, ≤18 years with 17.0% (617/3634), 19–35 years with 42.1% (1530/3634), 36–59 years with 38.1% (1386/3634), and ≥60 years with 2.8% (101/3634).

### 3.2. Overall Prevalence of *H*. *pylori* Infection (2015–2019)

The overall *H*. *pylori* prevalence between 2015 and 2019 was 35.7% (1298/3634) in the three clinics. Acacia Clinic contributed the highest proportion of 16.8% (611/1298), followed by City Centre Clinic (12.1% (439/1298)) and Bweyogerere Clinic (6.8% (248/1298)), respectively. Out of 1298 positive cases, 20.2% (736/1298) were males and 15.5% (562/1298) were females. Among the positive cases, age category of 19–35 years had the highest number of cases (16.6% (603/1298)), followed by 36–59 years with 13.2% (480/1298), then ≤18 years with 5.1% (187/1298), and lastly ≥60 years with 0.8% (28/1298).

### 3.3. Trend of the Prevalence of *H*. *pylori* Infection in Selected AAR Clinics

**Figure 3:**
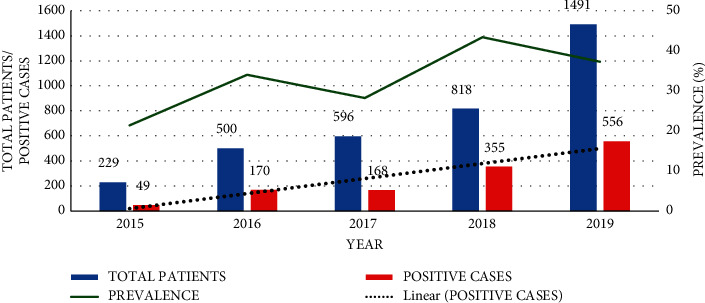
Annual trend of prevalence (%) of *H*. *pylori* infection at selected AAR clinics.

From year 2015 to 2019, the trends of *H*. *pylori* infection were increasing despite the fluctuation over the years. From Figure 3, the prevalence was 21.4% (49/229) in 2015, increased to 34.0% (170/500) in 2016, decreased to 28.2% (168/596) in 2017, then increased to 43.4% (355/818) in 2018, and finally declined to 37.3% (556/1491) in 2019.

**Figure 4:**
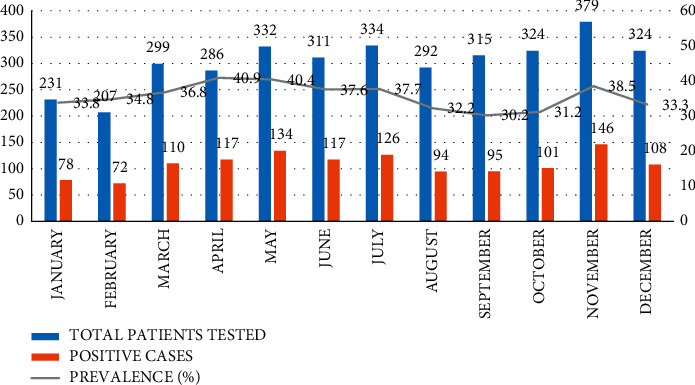
General temporal trends of *H*. *pylori* infection at AAR selected clinics.

Figure 4 shows overall monthly trends across all clinics; peak in prevalence was observed in March with 48.0% followed by April with 40.5% and May with 40.4%, and the lowest was obtained in September with 30.2%. However, sharp peaks were observed in prevalence in both March and November. The variations observed in the monthly prevalence of *H*. *pylori* at different clinics were not statistically significant.

### 3.4. Clinic-Specific *H*. *pylori* Prevalence

**Figure 5:**
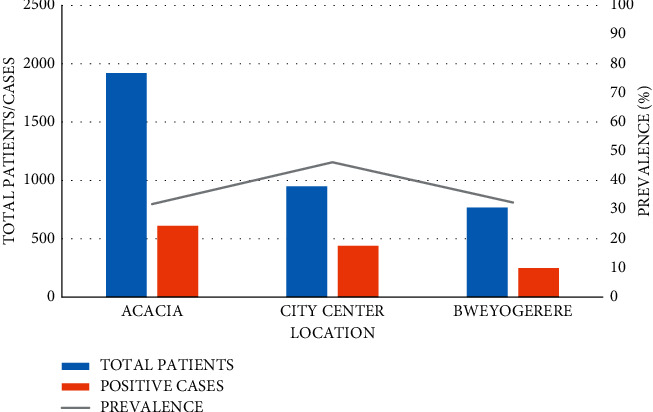
Clinic-specific overall performance.

From Figure 5, the highest prevalence was obtained from City Centre Clinic (46.2%), followed by Bweyogerere Clinic (32.4%) and Acacia Clinic (31.9%). Location was highly associated with the presence of *H*. *pylori* in the tested GIT patients.

Generally, City Centre Clinic had a higher annual prevalence compared to the other two clinics, that is, Acacia and Bweyogerere, over the years except in 2017 where all the clinics had almost the same prevalence (Bweyogerere = 28.2%, City Centre = 28.6%, and Acacia = 28.1%). GIT patients tested from City Centre Clinic were 1.84 times more likely to test positive for *H*. *pylori* than those tested from Acacia, and this was statistically significant (*p* < 0.001, 95% CI: 1.57–2.16). Although Bweyogerere Clinic was 1.02 times more likely to have positive cases compared to Acacia Clinic, this difference was statistically insignificant (*p*=0.794, 95% CI: 0.89–1.23) (Table 1).

The *H*. *pylori* prevalence varied with years, and the annual difference in *H*. *pylori* prevalence was statistically significant as compared to 2015; all the years had a *p* value <0.05, and 95% CIs were all above 1.0 (Table 1).

**Figure 6:**
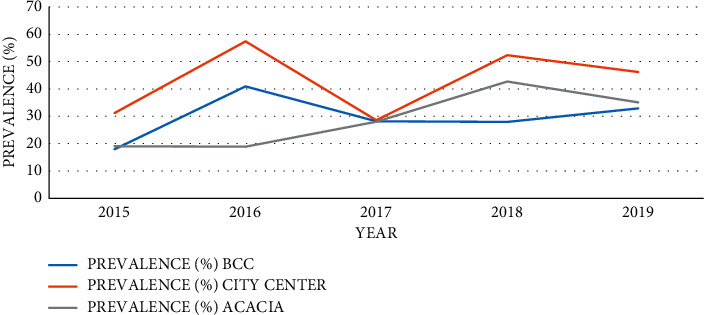
Clinic-specific annual trend of *H*. *pylori* infection (2015–2019).

Bweyogerere Clinic's highest prevalence was obtained in 2016, followed by 2019, 2017, 2018, and lastly 2015. In Acacia Clinic, the highest prevalence was reported in 2018, followed by 2019, 2017, 2015, and lastly 2016. In City Centre, 2016 reported the highest prevalence followed by 2018, 2019, 2015, and finally 2016 (Figure 6).

**Figure 7:**
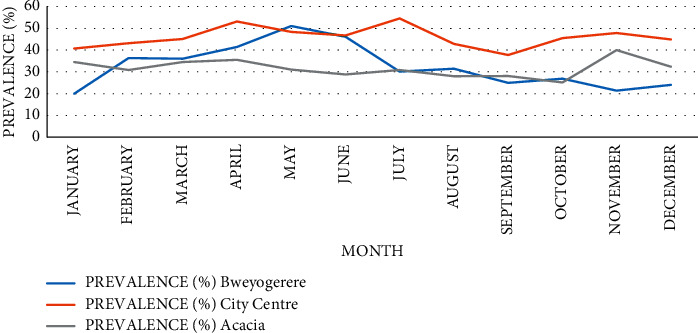
Clinic-specific temporal trend of *H*. *pylori* infection.

Figure 7 shows the monthly variations of *H*. *pylori* infection in different clinics, and the highest prevalence was recorded in different months in these clinics: City Centre in July, Acacia in November, and Bweyogerere in May. City Centre Clinic consistently reported higher monthly prevalence compared to other clinics except in May when Bweyogerere recorded the highest prevalence.

GIT patients tested from City Centre Clinic were 1.84 times more likely to test positive for *H*. *pylori* than those tested from Acacia, and this was statistically significant (OR = 1.84, *p* < 0.001, 95% CI: 1.57–2.16). The prevalence in males was 36.0%, slightly higher than that in females (35.4%); however, this difference was not statistically significant (OR = 0.97, *p*=0.680, 95% CI: 0.84–1.11).

The highest prevalence was observed in the age category of 19–35 years (39.4%) followed by 36–59 years (34.6%), under 18 years (30.3%), and finally ≥60 years (27.7%). It was noted that people between 19 and 35 years of age are 1.5 times more likely to be infected by *H*. *pylori* than those ≤18 years of age, and this was statistically significant (OR = 1.50, *p* < 0.001, 95% CI: 1.22–1.83). Patients between 36 and 59 years of age were 1.23 times more likely to be infected with this bacterium; however, this was not statistically significant (OR = 1.23, *p*=0.058, 95% CI = 0.99–1.49) (Table 1).

A multivariate model was developed adjusting for confounders. Sex and age were determined as risk factors through an iterative model building procedure as they gave the best model fit (*p* value = 0.5). As seen from Table 2, the presence of *H*. *pylori* was significantly affected by the age of the patient, especially those between 19 and 35 years (aOR = 1.49, *p* < 0.001, 95% CI = 1.22–1.82).

## 4. Discussion

*Helicobacter pylori* infection is extremely common worldwide with evidence from a recent systematic review suggesting that almost half of the world's population is infected [1, 2]. It has been documented that infection rates are higher in resource-poor settings and developing countries, with prevalence rates above 70% reported in Africa, the highest worldwide [2], whereas the prevalence in developed countries ranged between 25 and 40% [2, 8]. This is the first kind of study in Kampala carried out in a private clinic setting in the recent years.

The overall prevalence obtained from this study was 35.7% which is lower than 70% expected in African countries [2]. Similar studies in Ethiopia and Cameroon on a 5-year trend of *H*. *pylori* obtained a higher prevalence of 46.1% and 51.5%, respectively [25, 26]. The difference could be because of the variations in the study setting, that is, the previous two studies which were carried out in the primary care hospital used the *H*. *pylori* antibody screening (blood) test and considered a different study population, hence the higher prevalence.

The annual prevalence of 21.4% in 2015, 34.0% in 2016, 28.2% in 2017, 43.4% in 2018, and 37.3% in 2019 reported in this study was lower than the expected prevalence in Africa. This prevalence was lower than 87% among patients with stomach cancers and other cancers in Uganda at Mulago Hospital [17] and 60.5% among pregnant women at Kawempe Health Centre [19]. The high prevalence of 87% among patients with stomach cancers in Mulago Hospital could be due to the diagnostic technique used, that is, antibody detection from blood samples, which has a higher sensitivity than the stool antigen detection method and hence the elevated results. In addition, it is also published that *H*. *pylori* antibodies can be detected months later in blood even when there is no active infection [27].

The study prevalence was also lower than that executed in urban areas of other developing countries: 81.7% in Nigeria [28], 53.0% in Egypt [13], 64.39% in Cameroon [15], and 46.2% in Nairobi [29]. However, all these studies were carried out in the hospital setting, and others used endoscopy which is a different technique from the stool antigen test used in this study.

AAR clinic network serves both private and insured patients but mostly the latter. Therefore, the lower prevalence could be due to socioeconomic status of clients served at these clinics which is believed to be either middle or high. Studies concluded that the risk of acquiring *H*. *pylori* infection reduces with high socioeconomic status of the individuals [10, 30, 31].

The prevalence of *H*. *pylori* was fluctuating over the five years of this study: 21.4% in 2015, 34.0% in 2016, 28.2% in 2017, 43.4% in 2018, and finally 37.4% in 2019. Although the prevalence was fluctuating over a five-year period, it showed a general increase as shown in Figure 3. This is in contrary to similar studies in Ethiopia and Cameroon which illustrated a decreasing trend [25, 26]. The increase in the number of patients with GIT symptoms that seek medical services at these clinics over time can explain the increase in detected cases and hence the increasing trend of *H*. *pylori* infection as shown in Figure 4. This could also be due to awareness and increased application of the SAT technique by the medical personnel in AAR clinics. However, these data do not represent the general population of Kampala, but they suggest a significant burden of *H*. *pylori* in Kampala since it is densely populated with residents of various socioeconomic classes.

Monthly variation in prevalence observed at both general (Figure 4) and clinic-specific (Figure 7) levels was not statistically significant; therefore, there was no evidence that *H*. *pylori* is seasonal dependent. However, in Israel, a study conducted in dyspeptic patients deduced that the frequency of *H*. *pylori* infection significantly increased during the winter months and decreased in the summer (*p* < 0.007) [32]. The observed sharp peaks in March and November could be because most companies renew their insurance packages in March, while others expire in December. Therefore, employees tend to use their insurance maximally during these months.

This study showed that *H*. *pylori* infection in GIT patients is highly associated with location. GIT patients tested at City Centre Clinic were 1.838 times more likely to test positive for *H*. *pylori* compared to the ones tested at Acacia Clinic. A study in India showed that eating restaurant food is a predisposing factor to *H*. *pylori* [11]. Therefore, a higher prevalence of *H*. *pylori* infection reported in City Centre Clinic was probably because most of the patients served there were office people who often eat restaurant food. Since the *H*. *pylori* bacterium is majorly transmitted through either oral-to-oral or fecal-to-oral contact [33], therefore, sharing of feeding equipment (plates, cups, forks, among others) at workplace increases exposure, and eating food from common food suppliers could be a common source to all employees, hence the higher prevalence at this location.

With respect to gender, the relationship with *H*. *pylori* infections varies in the previous studies. Some studies show significantly higher *H*. *pylori* prevalence in males than females; in Ethiopia, males had 43.2% and females had 39.9% (*p*=0.002) [26], and others showed a higher prevalence in females though not statistically significant [14, 22]. In this study, there was a slight difference in gender prevalence (male: 36.0% and female: 35.3%); however, this difference was not statistically significant. The findings of this study were in agreement with other studies carried out in Nigeria, Lagos, *p*=0.315 [34], in Mwanza, Cameroon, *p*=0.901 [25], and in Ethiopia, *p*=0.746 [35]. Other studies also indicated a positive correlation between gender and *H*. *pylori* occurrence, in Kenya, *p*=0.003 [29], Ethiopia, *p*=0.002 [26, 10]. Variations in sample sizes, study settings such as hospitals (majority was hospitals and gastroenterology referral units), and testing techniques used could explain the difference in the findings.

A multivariate analysis found out that age was a predisposing factor for *H*. *pylori* infection (OR = 1.5, *p* < 0.001, 95% CI: 1.22–1.82). The infection was predominant in individuals between 19 and 35 years and 36 and 59 years with 39.4% and 34.6%, respectively, than young ones and elderly. This bacterium is said to be mostly acquired in childhood, especially when family members are infected, and it can last for a lifetime [36, 37].

Studies in Zimbabwe, Norway, Tanzania, and Iraq showed a significant increase of *H*. *pylori* infections with age [14, 38–40]. The prevalence was higher in patients of 16–45 years (30%) than in young ones, 1–15 years (8%), and elderly, 61–75 years (12%), in Iraq. In Norway, the prevalence of *H*. *pylori* increased with age: 0–11 years (0.6%), 12–17 years (20%), and above 18 years (45%). The elevated prevalence among middle-aged individuals could be due to stress and anxiety that are experienced during this stage of life caused by work, personal, financial, and family responsibilities as it was deduced that *H*. *pylori* occurrence was significantly associated with stress, anxiety, and depression (*p* < 0.001) [41].

The limitation of the study was that some data, especially about sociodemographics of the GIT patients, were missing from the database (Paras information system), for example, residence, source of food, source of drinking water, and occupation, which would give a better understanding as to why a certain location and age group had more infected clients.

## Figures and Tables

**Table 1:** Bivariate analysis of *H*. *pylori* infection versus demographics, years, and months.

Variable	Category	Seronegative, *n* (%)	Seropositive, *n* (%)	*p* value	OR	95% CI
Location	Acacia	1,307 (68.1)	611 (31.9)	Ref		
	City Centre	511 (53.8)	439 (46.2)	<0.001	1.84	1.57–2.16
	BC	518 (67.6)	248 (32.4)	0.794	1.02	0.86–1.23

Sex	Male	1,308 (64.0)	736 (36.0)	Ref		
	Female	1,028 (64.7)	562 (35.3)	0.680	0.97	0.84–1.11

Age category (years)	≤18	430 (69.7)	187 (30.3)	Ref		
	19–35	927 (60.6)	603 (39.4)	<0.001	1.50	1.22–1.83
	36–59	906 (65.4)	480 (34.6)	0.058	1.23	0.99–1.49
	≥60	73 (72.3)	28 (27.7)	0.599	0.44	0.55–1.49

Year	2015	180 (78.6)	49 (21.4)	Ref		
	2016	330 (66.0)	170 (34.0)	0.001	1.89	1.31–2.73
	2017	428 (71.8)	168 (28.2)	0.048	1.44	1.00–2.07
	2018	463 (56.6)	355 (43.4)	<0.001	2.82	2.00–3.98
	2019	935 (62.7)	556 (37.3)	<0.001	2.18	1.56–3.05

Month	January	153 (66.2)	78 (33.8)	Ref		
	February	135 (65.2)	72 (34.8)	0.030	0.67	0.49–0.96
	March	189 (63.2)	110 (36.8)	0.053	0.72	0.52–1.00
	April	169 (59.1)	117 (40.9)	0.168	0.77	0.53–1.12
	May	198 (59.6)	134 (40.4)	0.096	0.74	0.51–1.06
	June	194 (62.4)	117 (37.6)	0.418	0.88	0.63–1.21
	July	209 (62.3)	126 (37.7)	0.411	0.87	0.63–1.21
	August	198 (67.8)	94 (32.2)	0.307	0.84	0.60–1.17
	September	220 (69.8)	96 (30.2)	0.890	0.98	0.71–1.35
	October	223 (68.8)	101 (31.2)	0.533	0.91	0.66–1.24
	November	233 (61.5)	146 (38.5)	0.012	0.65	0.47–0.91
	December	216 (66.7)	108 (33.3)	0.006	0.62	0.44–0.88

**Table 2:** Multivariate logistic regression, epidemiological modelling.

Variable	Category	aOR	Std. err.	*Z*	*p* value (*P*)	95% confidence interval
Sex	Female	1.00	0.0683	−0.49	0.626	0.84–1.11
Age category	≤18 cons	0.44	0.04216	−8.56	0.000	0.37–0.53
	19–35	1.49	0.1524	3.93	<0.001	1.22–1.82
	36–59	1.21	0.1271	1.82	0.068	0.99–1.49
	≥60	0.88	0.2099	−0.55	0.585	0.55–1.40

## Data Availability

The datasets used and/or analyzed during the current study are available from the corresponding author upon reasonable request.

## Abbreviations

SDGs: Sustainable Development Goals
OR: Odds ratio
RR: Risk ratio
aOR: Adjusted odds ratio.
